# VianniaTopes: a database of predicted immunogenic peptides for *Leishmania* (*Viannia*) species

**DOI:** 10.1093/database/bay111

**Published:** 2018-10-25

**Authors:** Alejandro Llanes, Carlos Mario Restrepo, Ricardo Lleonart

**Affiliations:** 1 Instituto de Investigaciones Científicas y Servicios de Alta Tecnología (INDICASAT AIP), Ciudad del Saber, Panama City, Panama; 2 Department of Biotechnology, Acharya Nagarjuna University, Guntur, India

## Abstract

*Leishmania* is a protozoan parasite causing several disease presentations collectively known as leishmaniasis. Pathogenic species of *Leishmania* are divided into two subgenera, *L.* (*Leishmania*) and *L.* (*Viannia*). Species belonging to the *Viannia* subgenus have only been reported in Central and South America. These species predominantly cause cutaneous leishmaniasis, but in some cases, parasites can migrate to the nasopharyngeal area and cause a highly disfiguring mucocutaneous presentation. Despite intensive efforts, no effective antileishmanial vaccine is available for use in humans, although a few candidates mainly designed for *L.* (*Leishmania*) species are now in clinical trials. After sequencing the genome of *Leishmania panamensis*, we noticed a high degree of sequence divergence among several orthologous proteins from both subgenera. Consequently, some of the previously published candidates may not work properly for species of the *Viannia* subgenus. To help in vaccine design, we predicted CD4^+^ and CD8^+^ T cell epitopes in the theoretical proteomes of four strains belonging to the *Viannia* subgenus. Prediction was performed with at least two independent bioinformatics tools, using the most frequent human major histocompatibility complex (MHC) class I and class II alleles in the affected geographic area. Although predictions resulted in millions of peptides, relatively few of them were predicted to bind to several MHC alleles and can therefore be considered promiscuous epitopes. Comparison of our results to previous applications to species of the *Leishmania* subgenus confirmed that approximately half of the reported candidates are not present in *Viannia* proteins with a threshold of 80% sequence similarity and coverage. However, our prediction methodology was able to predict 70–100% of the candidates that could be found in *Viannia*. All the prediction data generated in this study are publicly available in an interactive database called VianniaTopes.

## Introduction

Pathogenic parasites of the *Leishmania* genus cause several disease presentations collectively known as leishmaniasis. Clinical cases have been reported in 98 countries, with more than 1 billion people living in affected areas (1). *Leishmania* parasites are transmitted to humans and animals by phlebotomine sandflies. The parasite exists in two major forms: promastigotes that develop within the insect vector and amastigotes that reside within macrophages of the vertebrate host. *Leishmania* species that are pathogenic to human are divided into two subgenera, *L.* (*Leishmania*) and *L.* (*Viannia*). Species belonging to the *Viannia* subgenus are distributed across Central and South America and predominantly cause cutaneous leishmaniasis. However, in some cases, the parasites can migrate to the nasopharyngeal area and cause mucocutaneous leishmaniasis (MCL), a more severe clinical presentation that is exclusive to this subgenus. The *Viannia* subgenus is broadly divided into two major species complexes, namely the *Leishmania braziliensis* species complex, which also includes *Leishmania peruviana*, and the *Leishmania guyanensis* species complex, which also includes *Leishmania panamensis*.

Traditional strategies to reduce the impact of leishmaniasis in affected areas have been mainly based on pharmacological treatment and epidemiological control (2). Pharmacological treatment has been hampered by the toxicity of available therapeutic drugs and the increasing problem of drug resistance in naturally occurring parasites (3). These limitations, together with the fact that epidemiological control is poor in many endemic countries, highlight the importance of developing effective vaccines against the parasite. Despite huge efforts, no effective antileishmanial vaccine is available to date for human use (4, 5). Although several vaccine candidates have shown positive results in mice and dogs, very few have entered phase I and II clinical trials. With several *Leishmania* genomes publicly available, one of the most promising alternatives nowadays is the identification of potential vaccine candidates at the genome-wide level (5). This approach, colloquially known as reverse vaccinology, requires a clear understanding of the cellular and molecular nature of the immune response against the parasite.

As with other intracellular parasites, immune response to *Leishmania* infection is highly dependent on the cellular component. Both CD4^+^ and CD8^+^ T cells are believed to be critical for disease control and long-term protection (6–8). A well-established model for *Leishmania major* infection in mice suggests that a CD4^+^ Th1 response helps control the primary infection and confers immunity, while a Th2 response favors disease progression (9). CD8^+^ T cells appear to play a key role in the establishment of the Th1 response and in subsequent immune protection through the production of IFN-γ, although their inherent cytotoxic activity may also be important (10). While this model appears to be valid for some species, significant differences have been reported when considering other species and the infection they cause in humans (11). For instance, it has been shown that species of the *Viannia* subgenus promote mixed Th1/Th2 hyperinflammatory responses in humans (12, 13). Similarly, a number of studies have highlighted the contradictory roles of CD8^+^ T cells in infection caused by *Viannia* species. Some studies have shown that exacerbated CD8^+^ activity with poorly regulated responses may be associated with disease progression and evolution to MCL (14), while finely regulated CD8^+^ responses are important for disease control in *L. braziliensis* (15, 16) and for immune protection against *L. panamensis* (17).

Due to the relevance of both CD4^+^ and CD8^+^ T cells in the immune response against *Leishmania*, computational prediction of potential antileishmanial vaccine candidates has focused on the identification of T cell epitopes. Such epitopes are peptides capable of binding to molecules of the major histocompatibility complex (MHC), MHC class I (MHC-I) for CD8^+^ T cells and MHC class II (MHC-II) in the case of CD4^+^ T cells. The first attempts to apply reverse vaccinology to pathogenic species of *Leishmania* focused on prediction of binding to murine MHC alleles (18, 19). Despite poor consensus among the programs, several of the predicted peptides were experimentally confirmed to be immunogenic. Furthermore, almost none of the best ranked peptides came from proteins known to be antigenic or tested as vaccine candidates in previous studies, thus highlighting the potential of genome screening for the identification of novel candidates. Improvement of bioinformatics methods further allowed the prediction of binding to human MHC (HLA) alleles. In order to reduce the number of proteins to which T cell epitope prediction is applied, researchers have typically focused on a pre-selected set of proteins, such as predicted membrane or surface-exposed proteins (20, 21), previously studied antigenic proteins (22), excreted/secreted proteins (23) or proteins overexpressed in the amastigote stage (24). The outcomes of these studies have been sets of best ranked peptides, predicted to bind to one or more human MHC alleles, some of which have been experimentally proven to be immunogenic *in vitro* or *in vivo*. However, all of these studies have mainly focused on Old World species of the *L.* (*Leishmania*) subgenus, with almost no consideration of New World species.

We have previously reported that several orthologous proteins from *L.* (*Leishmania*) and *L.* (*Viannia*) show high-sequence divergence, which in some cases resulted in the orthologs being considered different proteins (25). To the best of our knowledge, only one study has so far conducted T cell epitope prediction with human MHC alleles for a species of the *Viannia* subgenus (26). In this study, the authors screened the *L. braziliensis* theoretical proteome for peptides able to bind to several MHC-I and MHC-II alleles. A set of 10 undisclosed peptides were synthesized, and half of them were further found to be immunogenic *in vitro*. However, in this study, authors considered only peptides derived from surface proteins conserved among other *L.* (*Leishmania*) species, therefore potentially limiting the discovery of *Viannia*-specific epitopes. Currently, reference genomes are available for both *L. braziliensis* (27) and *L. panamensis* (25), making it possible to apply a reverse vaccinology strategy to identify potential vaccine candidates specific to this subgenus. Here we explored the proteins from four genomes of species belonging to the *Viannia* subgenus, in order to predict CD4^+^ and CD8^+^ T cell epitopes. Instead of reporting a list of best-ranked candidates, we stored all the positive predictions in a publicly available database named VianniaTopes. This solution allows researches not to restrict to a single set of pre-selected candidates that may be easily discarded in subsequent experimental evaluation. Instead, the database can be queried by using multiple criteria in order to add flexibility to selection schemes. The database is free and does not require the user to register in order to access it.

## Materials and methods

### Preparation of protein sequence data

We used the protein-coding genes annotated in the reference genomes for *L. braziliensis* (strain M2904) (27) and *L. panamensis* (strain PSC-1) (25), both downloaded from the Genomes resource of the National Center for Biotechnology Information. We also included the proteins from *L. braziliensis* strain M2903 and *L. panamensis* strain L13, downloaded from the TriTrypDB database (release 30) (28). Genes with unsequenced segments (gaps) and potential pseudogenes were subsequently discarded. OrthoMCL (29) was used to cluster the proteins from *L. braziliensis* strain M2903 and *L. panamensis* strains PSC-1 and L13 into the ortholog groups predefined in OrthoMCL-DB (version 5), which already contains the proteins from *L. braziliensis* strain M2904. Proteins left ungrouped from each genome were screened for sequence similarity and clustered into additional, putative *Viannia*-specific groups.

### Epitope prediction

Since T cell epitope predictions are highly dependent on MHC alleles, we first searched the dbMHC database (30) (currently available at ftp://ftp.ncbi.nlm.nih.gov/pub/mhc) for the most frequent MHC-I and MHC-II alleles in the geographical region of interest. Since very few allele frequency data were found for Central America, we selected the alleles with the highest frequencies in both South and North America. Only those alleles supported by all the predictive programs described below were further considered (Figure S1). The MHC-I alleles selected were HLA-A^*^02:01, HLA-A^*^24:02, HLA-A^*^31:01, HLA-A^*^68:01, HLA-B^*^15:01, HLA-B^*^39:01, HLA-B^*^40:02, HLA-C^*^03:03, HLA-C^*^04:01, HLA-C^*^07:02 and HLA-C^*^15:02. The MHC-II alleles selected were HLA-DRB1^*^08:02, HLA-DQA1^*^03:01, HLA-DQA1^*^04:01, HLA-DQA1^*^05:01, HLA-DQB1^*^03:01, HLA-DQB1^*^03:02, HLA-DQB1^*^04:02, HLA-DPA1^*^01:03 and HLA-DPB1^*^04:02. Alleles from the DP and DQ groups were used in the combinations HLA-DPA1^*^01:03/DPB1^*^04:02, HLA-DQA1^*^03:01/DQB1^*^03:02, HLA-DQA1^*^04:01/DQB1^*^04:02 and HLA-DQA1^*^05:01/DQB1^*^03:01, corresponding to pairs of α and β chains, respectively. Allele HLA-DRB1^*^08:02 was used separately, since α chains are not variable in the DR group.

Binding to MHC class I alleles was predicted for all the nonameric (9-mer) peptides derived from the proteins of interest by using two pan-specific methods, NetMHCpan v. 3.0 (31) and NetCTLpan (32), as well as the allele-specific Consensus method from the Immune Epitope Database (IEDB) MHC-I binding prediction tool (33). Potential epitopes were selected on the basis of the percentile rank (%Rank), with the cutoff values suggested by the authors for each program, i.e. %Rank <2.0 for NetMHCpan and %Rank <1.0 for both NetCTLpan and IEDB Consensus. In the case of NetMHCpan, authors also suggest a cutoff value of 0.5, below which peptides are predicted to be strong binders (SBs) to the corresponding alleles, while peptides with a %Rank equal or greater than 0.5 are considered to be weak binders.

Binding to MHC class II alleles was predicted for all the possible 15-mer peptides by using NetMHCIIpan v.3.1 (34) and the Consensus method of the IEDB MHC-II binding prediction tool (35, 36). As with MHC-I potential epitopes, the %Rank threshold suggested by the authors were used for selection (%Rank <10.0 for NetMHCIIpan and IEDB Consensus, %Rank <2.0 for NetMHCIIpan SBs).

Two major strategies were used to select the peptides according to epitope predictions, namely, considering only the peptides predicted by all the programs under the corresponding cut-off values (the intersection set) or considering all the peptides predicted by at least one program (the union set). An allele coverage was computed for each peptide under each selection scheme, calculated as the number of alleles for which the corresponding peptide was predicted to bind.

In order to predict peptides acting as combined MHC-I and MHC-II binders, all the selected 15-mer peptides were screened for overlapping 9-mer peptides previously predicted to act as MHC-I epitopes. For each 15-mer peptide with at least one overlapping MHC-I binder, a coverage of MHC-I alleles was computed in addition to its MHC-II allele coverage. This MHC-I allele coverage was computed as the nonredundant list of alleles to which all the corresponding overlapping 9-mer peptides were previously predicted to bind.

### Estimation of peptide properties

Several basic properties were also precomputed for each peptide to allow additional criteria useful for selection. Approximation methods were used to calculate physicochemical properties, such as the isoelectric point (pI) and charge at neutral pH (37), as well as solubility and hydrophobicity scores (38). Suitability of peptides for conventional synthesis was estimated empirically by taking into account several situations that are known to interfere with synthesis protocols, such as the presence of N-terminal glutamine or asparagine residues, three or more cysteine or methionine residues, two or more aspartate residues adjacent to glycine, proline or serine, as well as more than 80% of the sequence composed of β-sheet forming amino acids. Synthesis was considered to be potentially problematic for a peptide if its sequence meets two or more of the previous criteria.

### Sequence similarity searches and conservation analysis

BLAST (Basic Local Alignment Search Tool) (39) was used to align all the peptides to the proteins encoded in the human, mouse and *L. major* reference genomes. Several default options were adjusted to increase the sensitivity of BLAST searches performed with short input sequences. These include decreasing the word size to 2, increasing the expected value threshold to 20 000, using the PAM30 scoring matrix and deactivating compositional adjustments. Presence of the peptides in the theoretical proteome of the subject species was reported if query coverage and percent identity were both above 80%. The same strategy for alignment and selection thresholds were used when comparing our predicted peptides to those stored in the IEDB database.

### Database and web interface implementation

The VianniaTopes database was implemented in MySQL (MariaDB server v. 5.5) running on an Apache HTTP server in a 64-bit Linux system (Centos 7). MHC-I and MHC-II binding predictions for each peptide/allele pair were stored separately in the database and linked to the corresponding allele, peptide and protein tables by using a relational scheme (see Results). The web interface was designed by using conventional HTML and CSS styling, with the Perl 5 DBI and CGI modules for database interaction and web execution, respectively.

## Results

### Protein selection

A total of 33 870 proteins from the genomes of four strains belonging to the *Viannia* subgenus were selected for epitope prediction in this study. The selected proteins were clustered into 7624 ortholog groups, of which, 95 did not have representatives in other *L.* (*Leishmania*) species and are therefore potentially exclusive to the *Viannia* subgenus (Table S1). These groups were later used to select putative *Viannia*-specific epitopes (see Peptide properties and conservation analysis). Several proteins encoded by the genes in these groups are widely known to be critical for *Leishmania* virulence, and some of them are highly immunogenic. These include proteins involved in the synthesis of surface components such as the proteophosphoglycan, secreted proteases and membrane transporters.

**Figure 1:**
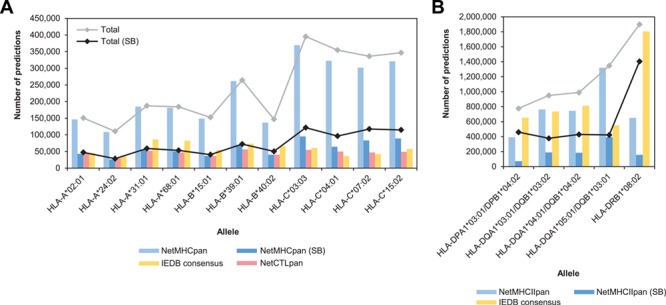
General statistics of T cell epitope predictions summarized by allele and predictive program for MHC-I **(A)** and MHC-II **(B)** alleles. Bars indicate the number of predictions per program expressed as peptide/allele pairs. Lines indicate the total number of predictions per allele, taking into account all the predictions from NetMHCpan/NetMHCIIpan or only the SB.

### Prediction of T cell epitopes

Epitope predictions were performed individually for each protein, using at least two predictive programs per MHC class. Since species of the *Viannia* subgenus have been exclusively reported in Central and South America, we intended to select the most frequent MHC alleles in both regions. However, due to the general lack of information regarding MHC allele frequencies in Central America, we selected those alleles that have a relatively high frequency and are present in both North and South America in the dbMHC database. In order to report comparable results, we further considered those alleles supported by all the predictive programs used for each MHC class, thus reducing the number of tested alleles to 11 MHC-I alleles and five combinations of nine MHC-II alleles (Figure S1).

Predictions were selected according to %Rank instead of binding affinity, since estimates of binding affinity are considered less reliable and are allele-dependent (40). Selection was done according to the %Rank cutoff values recommended by the authors, which for MHC-I prediction tools are between 1.0 and 2.0%. In order to cover all possible scenarios, we used two independent methods for clustering predictions, namely considering those peptide/allele pairs predicted by all the programs (the intersection set) or by at least one program (the union set). For MHC-I predictions, the union set has over 2.8 million peptide/allele pairs, while the intersection set includes 472 766 of these pairs (Figure S2). These numbers were respectively reduced to 802 545 and 196 892 when considering only the pairs with a NetMHCpan %Rank <0.5, colloquially known as SBs.

The total number of predictions per allele varied greatly among programs (Figure 1). The major source of this variation appears to be the number of NetMHCpan predictions, which was always higher than those from NetCTLpan and IEDB Consensus. However, the number of predictions appears to be roughly similar among the three programs when considering only the SB. It is also important to mention that the number of NetMHCpan predictions for HLA-C alleles was relatively higher than those observed for the rest of the alleles, although the difference is also less noticeable when considering only the SB.

The number of MHC-II binding predictions was always notably higher than the corresponding MHC-I predictions in each set, with nearly 6 million peptide/allele pairs in the union set and over 2.5 million in the intersection set (Figure S2). Since binding prediction is less reliable for MHC-II than for MHC-I, cutoff values recommended for %Rank are larger than those used for MHC-I, i.e. 10.0% for both NetMHCIIpan and IEDB Consensus and 2.0% for NetMHCIIpan SB. As expected, the number of SB was notably lower than the total number of binders predicted by NetMHCIIpan, but in this case, the number of IEDB Consensus predictions per alleles appears to be globally more similar to the latter (Figure 1B). Despite the lower number of alleles considered, we also observed variations in the number of predictions per allele, particularly for alleles HLA-DQA1^*^05:01/DQB1^*^03:01 and HLA-DRB1^*^08:02.

Since predictions were made by testing a peptide against several alleles, the same peptide can, and usually have, more than one positive prediction in the results, each one for a different allele. In order to group the results by peptide, instead of peptide/allele pairs, we counted the alleles to which each peptide was predicted to bind under each selection method (union or intersection). This measure was termed allele coverage and was considered to be a general indicator of how promiscuous peptides are in their capacity to bind to MHC alleles. Table 1 shows the nonredundant number of peptides computed for each category after summarizing across alleles.

**Table 1:** Nonredundant number of predicted peptides

**Selection method**	**MHC-I**	**MHC-II**
Union	1 437 008	3 516 121
Union (SB)	605 470	2 317 664
Intersection	218 376	1 702 834
Intersection (SB)	182 806	645 941

**Figure 2:**
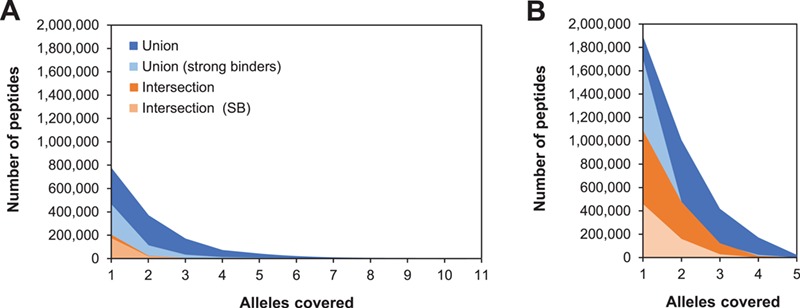
Number of peptides per allele coverage for selected MHC-I **(A)** and MHC-II **(B)** alleles. Plots show the nonredundant number of peptides predicted to bind two one or more alleles under each selection method. The same scale is used in both plots to emphasize the differences between both classes.

As expected, the nonredundant number of peptides in each category is always lower than the corresponding number of predictions, but the number of MHC-II binding peptides is also higher than that of MHC-I binding peptides. Regardless of the selection method, the number of peptides dramatically decreases with the increase in allele coverage (Figure 2). For MHC-I predictions, no peptide was found to have a 100% allele coverage either in the intersection or the union set. In the case of MHC-II predictions, only four peptides were concurrently predicted to cover all alleles (AAWAFVAALSRLLVC, ERFFSFMAAARARLE, SVAAWAFVAALSRLL and VAAWAFVAALSRLLV).

Additionally, 15-mer peptides were screened for overlapping 9-mers previously predicted to act as MHC-I epitopes, in order to identify peptides potentially capable to activate both CD4^+^ and CD8^+^ T cells. When using the union as the selection method, 3 052 839 15-mer peptides (87%) were found to have at least one overlapping MHC-I binding peptide. This number is reduced to 1 783 083 when considering only NetMHCpan and NetMHCIIpan SB. When using the intersection as selection method, the numbers are reduced to 924 085 and 809 810 for all the predictions or only the SB, respectively.

### Peptide properties and conservation analysis

Since the main goal of this work was the development of a database of predicted immunogenic peptides, we estimated several properties in addition to epitope predictions, which can help narrow the results of further selection strategies. These properties include molecular weight, pI, net charge, water solubility at neutral pH and suitability for conventional synthesis. It is important to mention that 30% of the peptides were predicted to have poor water solubility at neutral pH, a property that should be considered critical when selecting peptides to be synthesized for subsequent *in vitro* and *in vivo* assays. These estimations allow researchers to find a workaround to the experimental use of these peptides, such as changing the pH or coupling them to protein carriers.

To account for important taxonomic constraints in selection strategies, we also aligned all the predicted peptides to the mouse, human and *L. major* theoretical proteomes. A total of 81 515 (2%) and 71 683 (1%) peptides were found to share sequence similarity with human and mouse proteins, respectively, with query coverage and identity above 80%. On the contrary, 47% of all the predicted peptides exhibited sequence similarity with *L. major* proteins when using the previously mentioned thresholds. However, only 14% of the peptides were found to be present in *L. major* proteins with identical sequence. Nearly 6000 peptides with low-sequence similarity (<40%) to *L. major* proteins derive from potentially *Viannia*-specific ortholog groups reported in Table S1. Of these peptides, 44 were predicted to bind to more than one MHC-II allele and have at least one overlapping MHC-I peptide with comparable allele coverage (Table S2). These peptides can be considered as potentially promiscuous *Viannia*-specific epitopes.

### Database implementation

**Figure 3:**
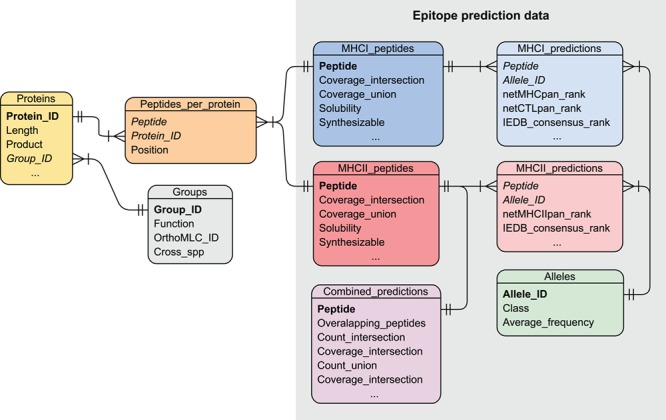
The entity-relationship diagram for the VianniaTopes database. Relationship among tables is represented by connecting lines, fields acting as primary keys are highlighted in bold and foreign keys are highlighted in italics.

The VianniaTopes database was implemented by using a relational model, normalized to the third normal form (Figure 3). In the VianniaTopes model, MHC binding predictions in the form of peptide/allele pairs were stored in separate tables and further linked to nonredundant tables of alleles and peptides. Peptide tables (MHCI_peptides and MHCII_peptides) store allele coverage values for each peptide under each selection scheme, as well as their taxonomic conservation and estimated values for physicochemical properties. These tables are also connected to an independent table (Peptides_per_protein) that indexes all the occurrences of each peptide in the sequences of the original proteins, which are in turn stored in the Proteins table. Consequently, this index table associates the peptide-based predictions with the proteins where the peptides appear. This association allows one to incorporate protein-related information in queries based on peptide properties, such as the presence of such peptides in proteins from ortholog groups conserved across the *Leishmania* genus or those sharing human or mouse orthologs.

**Figure 4:**
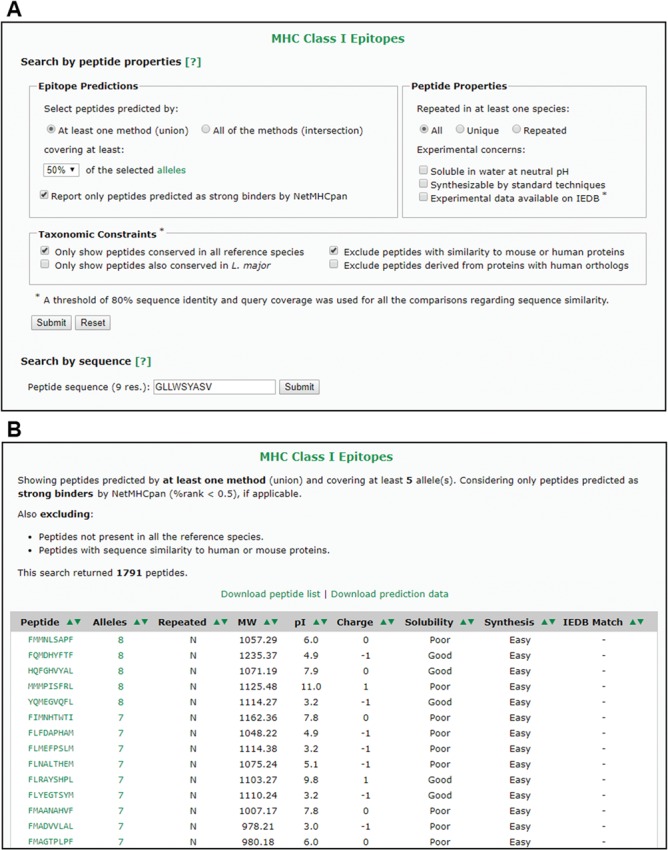
The VianniaTopes web interface. **(A)** Main search options for MHC-I prediction data. **(B)** Sample result for a search by peptide properties using the criteria shown in panel A.

The web interface allows users to separately search for peptides predicted to bind to MHC-I or MHC-II alleles, as well as the combined predictions. The database can be queried by peptide properties or by peptide sequence (Figure 4A). Queries based on peptide properties can be performed under one of the two proposed selection schemes (union or intersection) and can be constrained by several criteria, including allele coverage, peptide properties and taxonomic grouping. In all cases, the output of each search is interactive and can be sorted by different criteria or exported to text files with tab-separated values (Figure 4B).

### Validation

In order to validate our prediction methodology, we intended to compare our results with those of previous applications of reverse vaccinology to the *Leishmania* genus. However, this comparison was hampered by the fact that most of these studies focused on species belonging to the *L.* (*Leishmania*) subgenus, such as *L. major*, *Leishmania infantum* and *Leishmania donovani*. Consequently, many of the predicted peptides are not present with identical sequence in orthologous proteins from the *Viannia* subgenus due to sequence divergence. For instance, of the 34 peptides derived from the *L. major* theoretical proteome, suggested by Singh *et al.* (21) as optimal epitopes for the South American population, only five were found to be similar to proteins from species of the *Viannia* subgenus under the 80% threshold of query coverage and sequence identity. However, these five peptides were indeed selected by our methodology with comparable allele coverage (Table S3). Consequently, we first selected the peptides reported by four comprehensive studies that performed experimental validation of their results (Table 2). We then checked if those peptides that are present in the theoretical proteomes of *Viannia* species, with the previously mentioned similarity thresholds, were predicted by our methodology and are therefore present in the VianniaTopes database (Table S3).

**Table 2:** Comparison of results from this study with those of previous applications of reverse vaccinology to pathogenic species of *Leishmania*

	Seyed *et al.* (22)	Agallou *et al.* (41)	Naouar *et al.* (23)	Dikhit *et al.* (24)
**Total peptides**	18	145	78	37
**Present in *Viannia***	9 (50%)^a^	58 (40%)	35 (45%)	8 (22%)
**Present in VianniaTopes**	9 (100%)^b^	43 (74%)	24 (69%)	8 (100%)
**Not found in *Viannia***	9 (50%)^a^	87 (60%)	43 (55%)	29 (78%)

a ^a^Percentages referred to the total number of peptides.

b ^b^Percentages referred to the number of peptides found in *Viannia* proteins.

Seyed *et al.* (22) reported 18 peptides derived from six previously known *L. major* antigenic proteins. These peptides were then evaluated for binding to the HLA-A2 alleles most frequent in South West Asian populations. Nine of these peptides could be found in the *Viannia* theoretical proteomes and were also predicted by our methodology. Naouar *et al.* (23) reported 78 peptides derived from *L. major* excreted/secreted proteins as potential binders to allele HLA-A^*^02:01. Only 35 of these peptides could be found in *Viannia* proteins, and 24 of them were also predicted by our methodology as candidate binders to HLA-A^*^02:01. Dikhit *et al.* (24) suggested 37 peptides capable of binding to several MHC-II alleles, derived from proteins overexpressed in *L. donovani* amastigotes. Only eight of these peptides were found to be present in *Viannia* proteins, and all of them were predicted by our methodology as well. Agallou *et al.* (41) first screened proteins from *L. major* using murine MHC alleles. However, binding to human MHC-I and MHC-II alleles was predicted only for synthetic multi-epitope peptides designed later. Of the 145 initially reported peptides, only 58 were found in *Viannia* proteins, and 43 of these were predicted by our methodology.

Although these four studies performed experimental immunogenicity evaluation of the reported peptides, direct comparison with our results was not always possible, since the experimental assays were not necessarily performed for individual peptides. For instance, Seyed *et al.* (22) performed the experimental assays in pools of peptides, while Agallou *et al.* (41) used synthetic multi-epitope peptides. However, of the six peptides evaluated *in vitro* by Dikhit *et al.* (24), only peptide FDLFLFSNGAVVWWG was found to be present in *Viannia*, and it was also predicted by our methodology. Similarly, from the six peptides found to be immunogenic *in vitro* by Naouar *et al.* (23), peptides YLRTFPAAL and TLGVKQMVV were found in *Viannia*, although only the former was predicted by our methodology.

For a more comprehensive analysis, we compared our predicted peptides with those reported in studies conducted with *Leishmania* species in the IEDB database. This database stores results from only two studies that exclusively performed experimental assays with peptides derived from species of the *Viannia* subgenus, one in *L. braziliensis* (42) and the other one in *L. panamensis* (43). These studies collectively reported 14 peptides as potential T cell epitopes, all of which have associated prediction data in VianniaTopes (Table S4). However, there are more than 900 epitopes derived from other species of *Leishmania* in this database, many of which share sequence similarity with *Viannia* proteins under the previously mentioned similarity thresholds. Using these thresholds, we found that 2356 (MHC-I) and 2696 (MHC-II) peptides from VianniaTopes have associated experimental data on IEDB. Of which, 804 (MHC-I) and 757 (MHC-II) have positive results in T cell or MHC binding assays. The presence of experimental evidence in the IEDB database was incorporated into the corresponding peptide tables of VianniaTopes and was also included as a selection criterion on the web interface, for both MHC-I and MHC-II epitopes.

## Discussion

During the last decade, there have been several examples of application of reverse vaccinology to the pathogenic species of *Leishmania*. Most of these studies considered Old World species belonging to the *L.* (*Leishmania*) subgenus, with few applications to New World species and those belonging to the *L.* (*Viannia*) subgenus. Orthologous proteins from both subgenera may differ largely due to sequence divergence. This situation may cause that effective candidates designed for species belonging to the *L.* (*Leishmania*) subgenus could fail to induce a protective response against species from the *Viannia* subgenus, especially if they derive from highly divergent proteins. Also, the majority of the previous works has also focused in a limited set of proteins for epitope prediction, selected on the basis of previously reported immunogenicity, subcellular localization or overexpression in the amastigote stage. One of the very few studies that have included a species from the *Viannia* subgenus, *L. braziliensis*, narrowed the predictions to proteins shared with *L.* (*Leishmania*) species, thus potentially missing *Viannia*-specific candidates. Unfortunately, authors of this study did not publicly disclose the sequences of the selected peptides.

The main goal of this work was to identify potential CD4^+^ and CD8^+^ T cell epitopes by screening four genomes publicly available for species of the *Viannia* subgenus. The selected genomes cover two representative species from each of the two recognized species complexes of the *Viannia* subgenus, namely, *L. braziliensis* and *L. panamensis*. In order to avoid missing potentially good candidates, we did not discard candidates on the basis of properties and taxonomic distribution of the proteins from which they derive. Instead, all predictions meeting the selected cutoff values were stored in a publicly accessible database called VianniaTopes, intended to aid research groups working with the corresponding species. After several applications of the reverse vaccinology strategy to this and other pathogenic species, we have noticed that authors end up with relatively few candidates, whose number is reduced even more after performing experimental evaluation. In addition, candidates are sometimes filtered by properties that may not be as relevant as expected for protective immune responses against the corresponding pathogens, such as the use of B cell epitopes and the prioritization of surface-exposed or overexpressed proteins in *Leishmania* vaccine design (19). Availability of a database of unfiltered precomputed peptide/allele predictions allows researchers to choose among several properties of the peptides and their source proteins, in order to add flexibility to selection schemes customized according to their interests and priorities. This resource, however, does not pretend to compete with other comprehensive databases of experimentally characterized epitopes, such as IEDB. Instead, it is considered as a tool offering precomputed predictions made with a subset of alleles relevant to the geographic area that is affected by these particular parasites.

Predictions were performed by at least two independent programs per MHC class. MHC-I binding predictions were performed by using NetMHCpan, NetCTLpan and IEDB Consensus, while MHC-II predictions were done with NetMHCIIpan and IEDB Consensus. It is important to mention that NetCTLpan relies on an earlier version of NetMHCpan (v. 2.3), but also uses complementary predictions of proteasomal cleavage and efficiency of antigen transport, two critical steps in the MHC-I presentation pathway. The Consensus tool from IEDB, in turn, combines the results of several predictive methods for MHC-I and MHC-II binding, based on artificial neural networks, stabilized matrix methods and other machine learning strategies.

We observed several differences in the results, not only regarding individual peptide predictions, but also in the global number of predictions per allele. However, a relatively large degree of consensus among the programs allowed us to cluster the predictions in an intersection set, which includes peptides concurrently predicted by all programs in each class. To account for predictions for which there was partial or no consensus, we also computed a union set. Furthermore, suggested NetMHCpan and NetMHCIIpan cut-off values were used to classify the predicted peptides as strong and weak binders. In the case of MHC-I binding predictions, the number of SB is roughly similar to the number of binders predicted by the other two programs. Data from all the clustering sets were stored in the database and can be accessed by properly setting the search parameters in the user interface. In addition to the epitope prediction metrics such as rank and binding affinity, a relevant criterion for peptide selection in the database is allele coverage. This value is defined as the number of alleles to which a peptide was predicted to bind under each clustering set. Peptides capable of binding to several alleles, colloquially known as promiscuous, are expected to cover a larger portion of the target population and are therefore better candidates for a protective vaccine. As expected, the number of peptides markedly decreases as allele coverage increases, for both MHC-I and MHC-II alleles.

Although the primary goal of this work was to create a comprehensive database instead of suggesting a list of best-ranked candidates, we explored the number of peptides predicted to be promiscuous in proteins from ortholog groups that are potentially specific to the *Viannia* subgenus. We found 44 peptides deriving from such proteins that are capable of binding to more than one MHC-II alleles, with a similar coverage of MHC-I alleles when considering their overlapping 9-mers. Most of these potentially promiscuous peptides come from proteins whose function is still unknown, except for a phosphoglycan β-1,3-galactosyltransferase (LbrM.02.0240/LpmP.02.0140) and a glutathione peroxidase (LbrM.26.0810/LpmP.26.0780). Proteins with both of these functions are well-known virulence determinants in *Leishmania*. Curiously, LbrM.26.0810/LpmP.26.0780 has been recently identified as an additional gene encoding glutathione peroxidase in the genomes of *Viannia* species (25). As a potentially specific feature of the *Viannia* subgenus, this gene was implicated in the development of MCL and perhaps other atypical disease presentations.

Together with peptides coming from genes that are potentially *Viannia*-specific, we found over 6000 peptides with low similarity to *L. major* proteins. This finding evidences the high sequence divergence that exists in some orthologous proteins shared by the *L.* (*Leishmania*) and *L.* (*Viannia*) subgenera, despite a global conservation in gene content and order on their genomes. Due to this variation, we were not entirely able to compare our results with previous studies applying reverse vaccinology to pathogenic *Leishmania* species, all of which have mainly focused on the *L.* (*Leishmania*) subgenus. However, 70–100% of the peptides that could be found in *Viannia* were predicted by our methodology with similar metrics and allele coverage. The VianniaTopes database can be very helpful to research groups working with pathogenic *Viannia* species, which affect several countries of Central and South America. Peptides suspected to be *Viannia*-specific promiscuous epitopes can be evaluated as independent candidates or as members of multi-epitope constructions targeting a broader group of species or a larger geographic region.

## Supplementary Material

Supplementary Data

## Notes

**Database URL:** http://bioinfo.indicasat.org.pa/vianniatopes/index.html
