# Supplementary material for: VianniaTopes: a database of predicted immunogenic peptides for Leishmania (Viannia) species
Source: Database (Oxford). 2018 Oct 25;2018:bay111. doi: 10.1093/database/bay111 (PMC6201054; doi:10.1093/database/bay111)
Supplement: Supplementary Data [file bay111_suppl_information.pdf]

SUPPLEMENTARY INFORMATION

ViannniaTopes: a database of predicted immunogenic peptides for *Leishmania* (*Viannia*) species

Alejandro Llanes, Carlos Mario Restrepo, Ricardo Lleonart

Figure S1. Frequency of the selected MHC alleles in the target geographical region.

Figure S2. Venn diagrams showing the distribution of epitope predictions by MHC class, method and selection scheme.

Table S1. Genes in potentially *Viannia*-specific ortholog groups.

Table S2. Potentially promiscuous peptides predicted to act as MHC-I and MHC-II epitopes derived from proteins within Viannia-specific ortholog groups.

Table S3. Peptides from previously published studies applying reverse vaccinology strategy to pathogenic *Leishmania* species, used in the validation of our prediction methodology.

Table S4. Peptides with positive experimental results in IEDB, reported in studies exclusively conducted on *L. braziliensis* or *L. panamensis*.

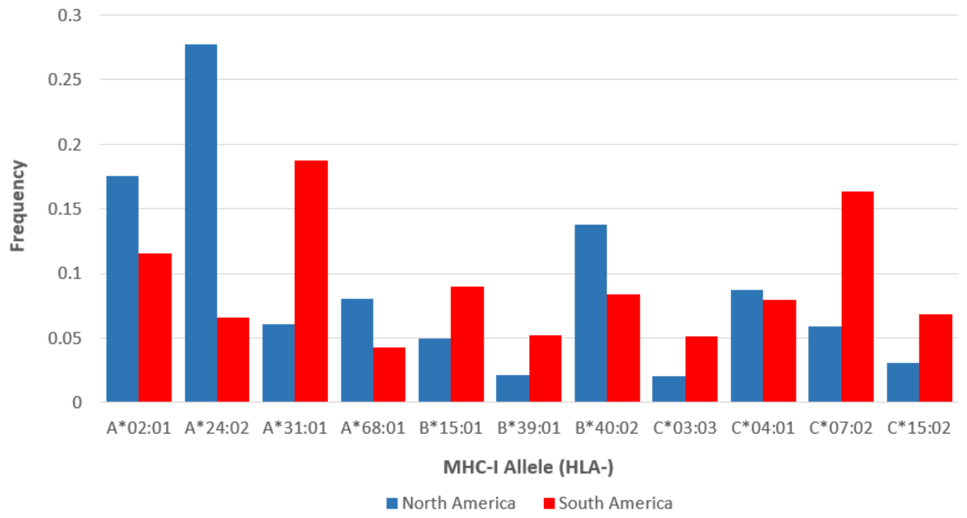

(A)

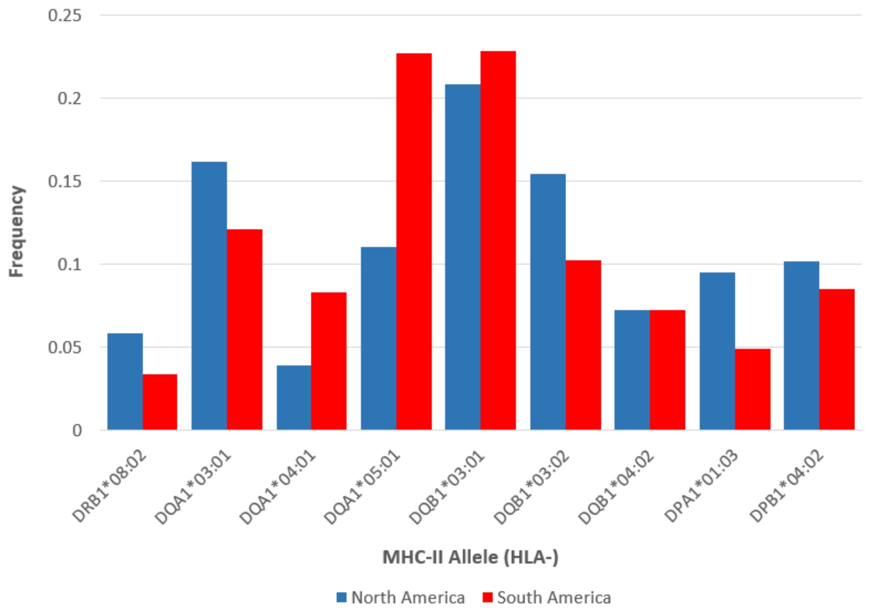

(B)

Figure S1. Frequency of the selected MHC alleles in the target geographical region. Figure shows the frequency of MHC-I **(A)** and MHC-II **(B)** alleles in the dbMHC database for North and South American populations.

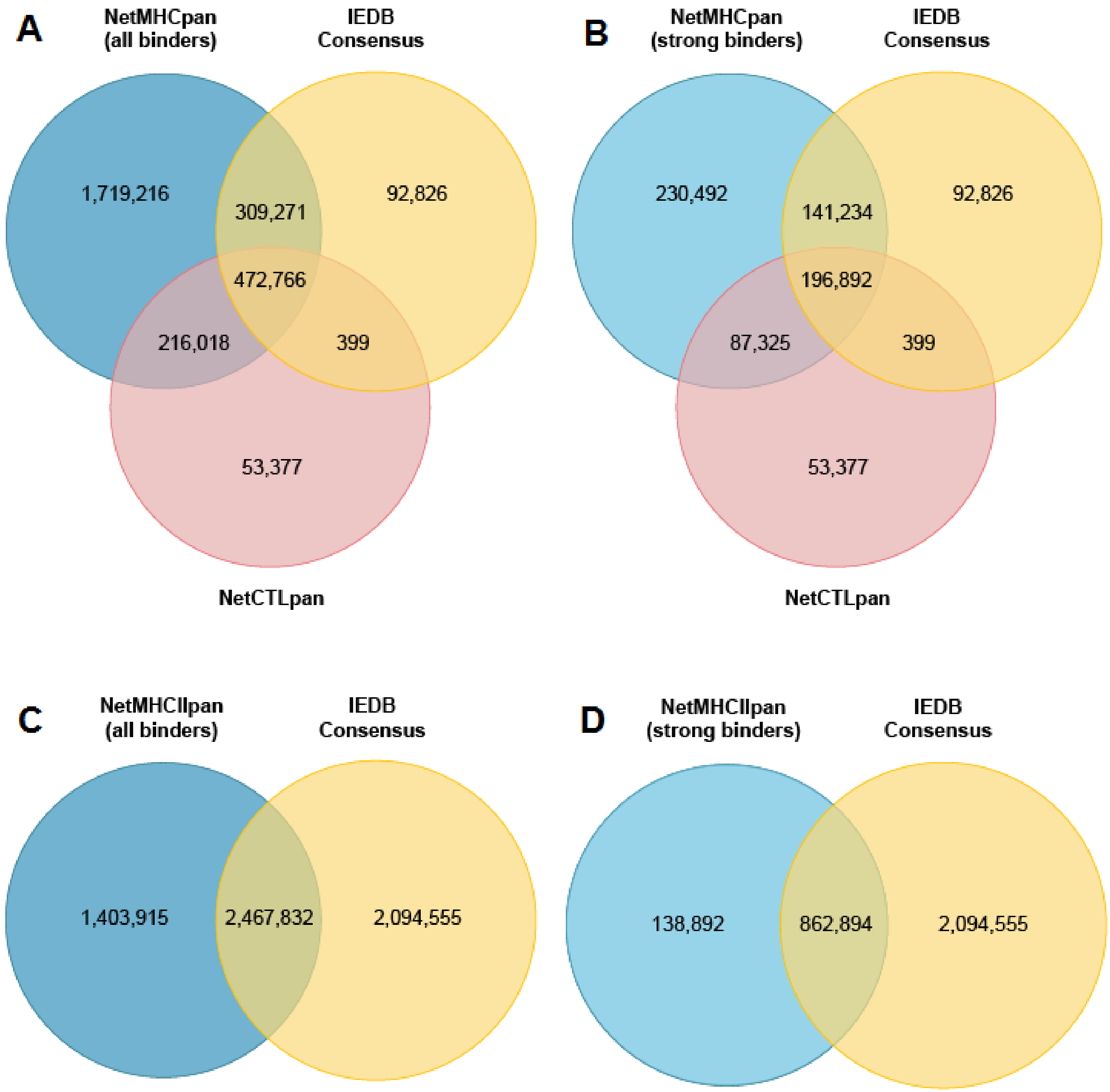

Figure S2. Venn diagrams showing the distribution of epitope predictions by MHC class, method and selection scheme. Figures show all the MHC-I binding predictions **(A)** and those with strong binders **(B)**, as well as all the MHC-II binding predictions **(C)** and those with strong binders **(D)**.

Table S1. Genes in potentially *Viannia*-specific ortholog groups.

Group ID	Putative function	Genes	Gene IDs
VIANNIA_0058	hypothetical protein	13	LBRM2903_070016100.1<br>LBRM2903_070016200.1<br>LBRM2903_250008700.1<br>LBRM2903_270035000.1<br>LBRM2903_290010400.1<br>LBRM2903_350047900.1<br>LBRM2903_350048000.1<br>LPAL13_000010400.1<br>LPAL13_000038200.1<br>LPAL13_000041600.1<br>LPAL13_010009100.1<br>LPAL13_070014100.1<br>LPAL13_290009500.1
VIANNIA_0471	amastin-like protein	7	LBRM2903_200018900.1<br>LBRM2903_200019000.1<br>LPAL13_200016200.1<br>LPAL13_200030900.1<br>LPAL13_200050300.1<br>LpmP.20.1620<br>LpmP.20.1640
VIANNIA_0472	beta-tubulin	6	LbrM.33.1010<br>LBRM2903_330013900.1<br>LBRM2903_330014000.1<br>LPAL13_000036800.1<br>LPAL13_210025500.1<br>LPAL13_330013200.1
VIANNIA_0765	phosphoglycan beta 1,3 galactosyltransferase	6	LbrM.02.0240<br>LBRM2903_020007400.1<br>LBRM2903_320048000.1<br>LPAL13_020006600.1<br>LPAL13_020007000.1<br>LpmP.02.0140
VIANNIA_0766	hypothetical protein	6	LbrM.25.0540<br>LBRM2903_150010200.1<br>LBRM2903_250014200.1<br>LPAL13_000040000.1<br>LPAL13_250012800.1<br>LpmP.25.0720
VIANNIA_0767	hypothetical protein	6	LbrM.31.2370<br>LBRM2903_230023600.1<br>LBRM2903_310033200.1<br>LPAL13_230021800.1<br>LPAL13_310026500.1<br>LpmP.31.1960
VIANNIA_0768	hypothetical protein	6	LbrM.32.0080<br>LBRM2903_200006800.1<br>LBRM2903_200034200.1<br>LBRM2903_320016700.1<br>LPAL13_000026400.1<br>LPAL13_200030500.1
VIANNIA_0769	ABC transporter, putative	5	LbrM.33.1550<br>LBRM2903_330021200.1<br>LPAL13_000053800.1<br>LPAL13_010008000.1<br>LPAL13_330019400.1

VIANNIA_0770	hypothetical protein	5	LBRM2903_310035700.1<br>LBRM2903_310035800.1<br>LPAL13_310028900.1<br>LPAL13_310029000.1<br>LpmP.31.2180
VIANNIA_1594	unspecified product	5	LbrM.23.0770<br>LBRM2903_230016400.1<br>LBRM2903_230016500.1<br>LPAL13_110009000.1<br>LPAL13_110009100.1
VIANNIA_1595	beta tubulin-like protein	5	LBRM2903_010014400.1<br>LBRM2903_010014500.1<br>LPAL13_000027000.1<br>LPAL13_010012500.1<br>LpmP.01.0810
VIANNIA_1596	cysteine peptidase, Clan CA, family C2, putative	4	LbrM.20.5390<br>LBRM2903_200068700.1<br>LBRM2903_200068800.1<br>LPAL13_200060400.1
VIANNIA_1597	amastin-like surface protein-like protein	4	LbrM.24.1280<br>LBRM2903_240018900.1<br>LPAL13_240018700.1<br>LPAL13_330042500.1
VIANNIA_1598	alpha tubulin	4	LbrM.29.2700<br>LBRM2903_130007300.1<br>LBRM2903_130007400.1<br>LBRM2903_290035900.1
VIANNIA_7163	hypothetical protein	4	LbrM.06.1180<br>LBRM2903_060019200.1<br>LPAL13_060018200.1<br>LpmP.06.1180
VIANNIA_7164	amastin-like protein	4	LbrM.08.0680<br>LBRM2903_290021100.1<br>LPAL13_080010300.1<br>LpmP.29.1390
VIANNIA_7165	hypothetical protein	4	LbrM.11.1130<br>LBRM2903_110019600.1<br>LPAL13_110017700.1<br>LpmP.11.1320
VIANNIA_7166	hypothetical protein	4	LBRM2903_130014200.1<br>LBRM2903_130014300.1<br>LPAL13_100012100.1<br>LPAL13_130013500.1
VIANNIA_7167	Carboxypeptidase Taq (M32) metallopeptidase, putative	4	LBRM2903_140006700.1<br>LBRM2903_140006800.1<br>LPAL13_140006600.1<br>LPAL13_140006700.1
VIANNIA_7168	hypothetical protein, conserved	4	LbrM.16.1060<br>LBRM2903_160017700.1<br>LPAL13_160014500.1<br>LpmP.16.0990
VIANNIA_7169	hypothetical protein	4	LbrM.20.3580<br>LBRM2903_200050000.1<br>LPAL13_200043300.1<br>LpmP.20.3720
VIANNIA_7170	hypothetical protein	4	LbrM.20.5420<br>LBRM2903_200069100.1<br>LPAL13_200060800.1<br>LpmP.20.5390
VIANNIA_7171	hypothetical protein	4	LbrM.22.1470<br>LBRM2903_220021500.1<br>LPAL13_220018200.1<br>LpmP.22.1460

VIANNIA_7172	hypothetical protein	4	LbrM.26.0010<br>LBRM2903_260005000.1<br>LPAL13_260005000.1<br>LpmP.26.0010
VIANNIA_7173	glutathione peroxidase-like protein, putative	4	LbrM.26.0810<br>LBRM2903_260012900.1<br>LPAL13_260012600.1<br>LpmP.26.0780
VIANNIA_7174	histone H1-like protein	4	LbrM.27.1740<br>LBRM2903_270023100.1<br>LPAL13_270023200.1<br>LpmP.27.1600
VIANNIA_7175	hypothetical protein	4	LBRM2903_290019800.1<br>LBRM2903_290019900.1<br>LPAL13_290017200.1<br>LPAL13_290017300.1
VIANNIA_7176	hypothetical protein	4	LbrM.30.0010<br>LBRM2903_300005100.1<br>LPAL13_300005100.1<br>LpmP.30.0010
VIANNIA_7177	multidrug resistance protein, copy 1-like protein	4	LbrM.31.1520<br>LBRM2903_310017900.1<br>LPAL13_310013600.1<br>LpmP.31.1210
VIANNIA_7178	beta-galactofuranosyltransferase-like protein	4	LbrM.33.0310<br>LBRM2903_330008000.1<br>LPAL13_330008000.1<br>LpmP.33.0350
VIANNIA_7179	hypothetical protein, conserved	4	LbrM.33.1340<br>LBRM2903_330018400.1<br>LPAL13_330017000.1<br>LpmP.33.1190
VIANNIA_7180	hypothetical protein	4	LbrM.33.3180<br>LBRM2903_330039700.1<br>LPAL13_330038000.1<br>LpmP.33.3020
VIANNIA_7181	hypothetical protein	4	LbrM.34.2010<br>LBRM2903_340027000.1<br>LPAL13_340025400.1<br>LpmP.34.1930
VIANNIA_7182	hypothetical protein	4	LBRM2903_350077300.1<br>LBRM2903_350077400.1<br>LPAL13_350072700.1<br>LPAL13_350072800.1
VIANNIA_7183	hypothetical protein, conserved	3	LBRM2903_040011800.1<br>LBRM2903_040012100.1<br>LPAL13_040012200.1
VIANNIA_7184	hypothetical protein	3	LBRM2903_080014100.1<br>LBRM2903_080014200.1<br>LPAL13_000034300.1
VIANNIA_7185	GP63, leishmanolysin (LbrM.10.1540.1)	3	LbrM.10.1540<br>LBRM2903_000009300.1<br>LBRM2903_000009400.1
VIANNIA_7186	hypothetical protein	3	LbrM.23.0550<br>LBRM2903_230011600.1<br>LPAL13_230011200.1
VIANNIA_7187	heat-shock protein hsp70, putative	3	LbrM.28.2970<br>LBRM2903_280037200.1<br>LPAL13_110015300.1
VIANNIA_7188	hypothetical protein, conserved	3	LbrM.31.2340<br>LBRM2903_310032500.1<br>LPAL13_310026000.1

VIANNIA_7189	d-xylulose reductase, putative	3	LbrM.33.0510<br>LBRM2903_330010300.1<br>LBRM2903_330010400.1
VIANNIA_7190	hypothetical protein, conserved	3	LbrM.34.3390<br>LBRM2903_340042700.1<br>LBRM2903_350032900.1
VIANNIA_7191	hypothetical protein	3	LBRM2903_350079000.1<br>LPAL13_350074300.1<br>LpmP.11.0520
VIANNIA_7192	hypothetical protein	3	LBRM2903_200075300.1<br>LPAL13_200066500.1<br>LpmP.12.1040
VIANNIA_7193	hypothetical protein	3	LBRM2903_130019300.1<br>LPAL13_130018500.1<br>LpmP.13.1380
VIANNIA_7194	hypothetical protein	3	LBRM2903_140010900.1<br>LPAL13_140010700.1<br>LpmP.14.0550
VIANNIA_7195	hypothetical protein	3	LBRM2903_200012200.1<br>LPAL13_200010500.1<br>LpmP.20.0630
VIANNIA_7196	hypothetical protein	3	LBRM2903_320034800.1<br>LPAL13_320032600.1<br>LpmP.32.2640
VIANNIA_7197	hypothetical protein	3	LBRM2903_330043500.1<br>LPAL13_330041700.1<br>LpmP.33.3330
VIANNIA_7326	hypothetical protein, conserved	3	LbrM.04.0590<br>LBRM2903_040011000.1<br>LPAL13_040011300.1
VIANNIA_7327	ubiquitin-fusion protein	3	LbrM.09.0640<br>LBRM2903_090015000.1<br>LPAL13_000026200.1
VIANNIA_7328	aminopeptidase, putative	3	LbrM.11.0400<br>LBRM2903_110008800.1<br>LpmP.11.0640
VIANNIA_7329	hypothetical protein, conserved	3	LbrM.21.2130<br>LBRM2903_080018200.1<br>LpmP.08.1070
VIANNIA_7330	hypothetical protein	3	LbrM.22.1380<br>LBRM2903_220020400.1<br>LpmP.22.1370
VIANNIA_7331	hypothetical protein	3	LBRM2903_240022000.1<br>LPAL13_240019900.1<br>LPAL13_240020000.1
VIANNIA_7332	hypothetical protein	3	LbrM.25.0570<br>LBRM2903_250014800.1<br>LPAL13_250013400.1
VIANNIA_7333	ABC transporter, putative	3	LbrM.27.1060<br>LBRM2903_270016500.1<br>LPAL13_270016800.1
VIANNIA_7334	hypothetical protein	3	LbrM.31.0200<br>LBRM2903_310006200.1<br>LpmP.29.1810
VIANNIA_7335	hypothetical protein, conserved	3	LbrM.31.1000<br>LBRM2903_310017300.1<br>LpmP.31.0810
VIANNIA_7336	hypothetical protein	3	LbrM.34.1660<br>LBRM2903_340022900.1<br>LpmP.34.1600

VIANNIA_7337	hypothetical protein, conserved	3	LBRM2903_340045900.1<br>LPAL13_340042700.1<br>LPAL13_340042800.1
VIANNIA_7338	BT1 family, putative	3	LBRM2903_340061500.1<br>LPAL13_320009700.1<br>LPAL13_340057800.1
VIANNIA_7339	phosphoglycan beta 1,3 galactosyltransferase-like protein	2	LbrM.02.0790<br>LBRM2903_020007500.1
VIANNIA_7340	BT1 family, putative	2	LBRM2903_040005200.1<br>LBRM2903_040005300.1
VIANNIA_7341	ATP synthase F1, alpha subunit, putative (fragment)	2	LbrM.05.0510<br>LBRM2903_050010300.1
VIANNIA_7342	hypothetical protein, conserved	2	LBRM2903_080013900.1<br>LPAL13_080010400.1
VIANNIA_7343	hypothetical protein, conserved	2	LBRM2903_110009300.1<br>LBRM2903_110009400.1
VIANNIA_7344	protein associated with differentiation 8, putative	2	LbrM.13.1550<br>LBRM2903_130021700.1
VIANNIA_7345	P-type H+ATPase, putative	2	LbrM.18.1730<br>LBRM2903_000009600.1
VIANNIA_7346	hypothetical protein, unknown function	2	LbrM.19.1900<br>LBRM2903_190026200.1
VIANNIA_7347	hypothetical protein, unknown function	2	LbrM.19.1920<br>LPAL13_040012000.1
VIANNIA_7348	amastin-like surface protein, putative	2	LbrM.20.0950<br>LBRM2903_200016500.1
VIANNIA_7349	pumilio protein 9, putative	2	LbrM.20.5610<br>LPAL13_200062600.1
VIANNIA_7350	orthologous HASP	2	LbrM.23.1110<br>LPAL13_230018700.1
VIANNIA_7351	hypothetical protein	2	LbrM.29.2910<br>LBRM2903_340005400.1
VIANNIA_7352	histone H4	2	LbrM.30.0450<br>LBRM2903_300009500.1
VIANNIA_7353	sodium stibogluconate resistance protein, putative	2	LbrM.31.1140<br>LBRM2903_310019100.1
VIANNIA_7354	adenosine deaminase-like protein	2	LbrM.33.0250<br>LBRM2903_310012500.1
VIANNIA_7355	hypothetical protein, conserved	2	LbrM.35.7400<br>LPAL13_000009700.1
VIANNIA_7356	E2 ubiquitin ligase-like protein	2	LPAL13_090014900.1<br>LpmP.09.1010
VIANNIA_7357	hypothetical protein	2	LPAL13_200015200.1<br>LpmP.20.1070
VIANNIA_7358	hypothetical protein	2	LPAL13_010010400.1<br>LPAL13_240012600.1
VIANNIA_7359	PIN domain containing protein, putative	2	LBRM2903_310032800.1<br>LPAL13_310026200.1
VIANNIA_7360	L-ribulokinase, putative	2	LBRM2903_350006400.1<br>LPAL13_350005200.1
VIANNIA_7456	hypothetical protein	2	LbrM.11.1100<br>LpmP.11.1290
VIANNIA_7457	surface antigen protein, putative	2	LbrM.12.0760<br>LpmP.12.0760
VIANNIA_7458	hypothetical protein, conserved	2	LBRM2903_140007800.1<br>LPAL13_140007700.1
VIANNIA_7459	hypothetical protein	2	LBRM2903_210024400.1<br>LPAL13_210022700.1
VIANNIA_7460	oxidoreductase-like protein	2	LBRM2903_230012500.1<br>LPAL13_230012100.1
VIANNIA_7461	adenine phosphoribosyltransferase	2	LbrM.26.0120<br>LBRM2903_260006200.1

VIANNIA_7462	hypothetical protein, conserved	2	LbrM.28.1800<br>LpmP.28.1740
VIANNIA_7463	FAD binding domain containing protein, putative	2	LBRM2903_290008500.1<br>LPAL13_290007900.1
VIANNIA_7464	S1/P1 Nuclease, putative	2	LBRM2903_310035200.1<br>LPAL13_310028200.1
VIANNIA_7465	hypothetical protein, conserved	2	LBRM2903_310041300.1<br>LPAL13_310034400.1
VIANNIA_7466	hypothetical protein	2	LbrM.35.6670<br>LpmP.35.6510

Table S2. Potentially promiscuous peptides predicted to act as MHC-I and MHC-II epitopes derived from proteins within *Viannia*-specific ortholog groups.

Peptide[1]	MHCII Coverage	MHCII Alleles	Overlapping 9-mers	MHCI Coverage	MHCI Alleles	Proteins
**VIANNIA_0765 (phosphoglycan beta-1,3-galactosyltransferase)**						
AMHAINASAAMLQRA<br>DAMHAINASAAMLQR	4	DQA1*03:01/DQB1*03:02<br>DQA1*04:01/DQB1*04:02<br>DQA1*05:01/DQB1*03:01<br>DRB1*08:02	MHAINASAA<br>HAINASAAM<br>AINASAAML<br>NASAAMLQR	7	A*68:01<br>B*15:01<br>B*39:01<br>C*03:03<br>C*04:01<br>C*07:02<br>C*15:02	LBRM2903_020007400.1<br>LPAL13_020007000.1<br>LbrM.02.0240<br>LpmP.02.0140
ATYATAIAATRDLVP<br>DTATYATAIAATRDL<br>TATYATAIAATRDLV	4	DQA1*03:01/DQB1*03:02<br>DQA1*04:01/DQB1*04:02<br>DQA1*05:01/DQB1*03:01<br>DRB1*08:02	ATYATAIAA<br>YATAIAATR<br>TAIAATRDL	4	A*31:01<br>A*68:01<br>C*03:03<br>C*15:02	
**VIANNIA_0767 (hypothetical protein)**						
NWMSSSIELEAVDVR<br>WMSSSIELEAVDVRK	3	DPA1*03:01/DPB1*04:02<br>DQA1*03:01/DQB1*03:02<br>DQA1*04:01/DQB1*04:02	NWMSSSIEL<br>MSSSIELEA<br>SSSIELEAV<br>ELEAVDVRK	6	A*24:02<br>A*68:01<br>B*39:01<br>C*04:01<br>C*07:02<br>C*15:02	LBRM2903_310033200.1<br>LPAL13_310026500.1<br>LbrM.31.2370<br>LpmP.31.1960
**VIANNIA_7165 (hypothetical protein)**						
EGIGWYGAISVVDVL<br>GIGWYGAISVVDVLI<br>IGWYGAISVVDVLIG	4	DQA1*03:01/DQB1*03:02<br>DQA1*04:01/DQB1*04:02<br>DQA1*05:01/DQB1*03:01<br>DRB1*08:02	IGWYGAISV<br>YGAISVVDV<br>GAISVVDVL	2	C*03:03<br>C*15:02	LBRM2903_110019600.1<br>LPAL13_110017700.1<br>LbrM.11.1130<br>LpmP.11.1320
**VIANNIA_7170 (hypothetical protein)**						
MGDSAVLLEEHTAVL	2	DQA1*03:01/DQB1*03:02<br>DQA1*04:01/DQB1*04:02	VLLEEHTAV<br>LLEEHTAVL	6	A*02:01<br>B*39:01<br>C*03:03<br>C*04:01<br>C*07:02<br>C*15:02	LBRM2903_200069100.1<br>LPAL13_200060800.1<br>LbrM.20.5420<br>LpmP.20.5390

**VIANNIA_7171 (hypothetical protein)**						
ATMSEAALVFESTMA<br>MSEAALVFESTMAEG<br>FRTATMSEAALVFES<br>RTATMSEAALVFEST<br>SEAALVFESTMAEGM<br>TMSEAALVFESTMAE	3	DQA1*03:01/DQB1*03:02<br>DQA1*04:01/DQB1*04:02<br>DQA1*05:01/DQB1*03:01	ATMSEAALV<br>TMSEAALVF<br>SEAALVFES<br>AALVFESTM<br>FRTATMSEA<br>TATMSEAAL	9	A*02:01<br>A*24:02<br>B*15:01<br>B*39:01<br>B*40:02<br>C*03:03<br>C*04:01<br>C*07:02<br>C*15:02	LBRM2903_220021500.1<br>LPAL13_220018200.1<br>LbrM.22.1470<br>LpmP.22.1460
**VIANNIA_7173 (glutathione peroxidase-like protein putative)**						
APGASIAEIDVRIEE<br>ARFAPGASIAEIDVR<br>ASIAEIDVRIEELLH<br>FAPGASIAEIDVRIE<br>GASIAEIDVRIEELL<br>PGASIAEIDVRIEEL<br>PYARFAPGASIAEID<br>RFAPGASIAEIDVRI<br>YARFAPGASIAEIDV	3	DQA1*03:01/DQB1*03:02<br>DQA1*04:01/DQB1*04:02<br>DQA1*05:01/DQB1*03:01	SIAEIDVRI<br>ARFAPGASI<br>RFAPGASIA	5	A*02:01<br>B*39:01<br>C*04:01<br>C*07:02<br>C*15:02	LBRM2903_260012900.1<br>LPAL13_260012600.1<br>LbrM.26.0810<br>LpmP.26.0780
MLDATVATVEDEDLT<br>SMLDATVATVEDEDL<br>SVSMLDATVATVEDE<br>VSMLDATVATVEDED<br>VSVSMLDATVATVED	3	DQA1*03:01/DQB1*03:02<br>DQA1*04:01/DQB1*04:02<br>DQA1*05:01/DQB1*03:01	MLDATVATV<br>SMLDATVAT<br>VSMLDATVA<br>SVSMLDATV	5	A*02:01<br>B*39:01<br>C*03:03<br>C*04:01<br>C*15:02	
**VIANNIA_7176 (hypothetical protein)**						
DLAAISVAQERILLQ<br>LAAISVAQERILLQE<br>VDLAAISVAQERILL<br>PVDLAAISVAQERIL	5	DPA1*03:01/DPB1*04:02<br>DQA1*03:01/DQB1*03:02<br>DQA1*04:01/DQB1*04:02<br>DQA1*05:01/DQB1*03:01<br>DRB1*08:02	AAISVAQER<br>ISVAQERIL<br>SVAQERILL	4	A*31:01<br>A*68:01<br>C*03:03<br>C*15:02	LBRM2903_300005100.1<br>LPAL13_300005100.1<br>LbrM.30.0010<br>LpmP.30.0010
GLLWSYASVVFDSSL<br>LLWSYASVVFDSSLV<br>LWSYASVVFDSSLVF<br>RGLLWSYASVVFDSS	5	DPA1*03:01/DPB1*04:02<br>DQA1*03:01/DQB1*03:02<br>DQA1*04:01/DQB1*04:02<br>DQA1*05:01/DQB1*03:01<br>DRB1*08:02	GLLWSYASV<br>LLWSYASVV<br>LWSYASVVF<br>ASVVFDSSL<br>SVVFDSSLV<br>VVFDSSLVF	7	A*02:01<br>A*24:02<br>B*15:01<br>C*03:03<br>C*04:01<br>C*07:02<br>C*15:02	

**VIANNIA_7180 (hypothetical protein)**						
EDQVVAFPQEQPTCL	2	DQA1*03:01/DQB1*03:02<br>DQA1*04:01/DQB1*04:02	FPQEQPTCL	3	B*39:01<br>C*03:03<br>C*04:01	LBRM2903_330039700.1<br>LPAL13_330038000.1<br>LbrM.33.3180<br>LpmP.33.3020
**VIANNIA_7181 (hypothetical protein)**						
GVEEIALWLEVYQAV<br>RGVEEIALWLEVYQA	3	DPA1*03:01/DPB1*04:02<br>DQA1*03:01/DQB1*03:02<br>DQA1*04:01/DQB1*04:02	EEIALWLEV<br>ALWLEVYQA<br>LWLEVYQAV	3	A*02:01<br>A*24:02<br>B*40:02	LbrM.34.2010<br>LBRM2903_340027000.1<br>LPAL13_340025400.1<br>LpmP.34.1930
LRGVEEIALWLEVYQ<br>SLRGVEEIALWLEVY	3	DPA1*03:01/DPB1*04:02<br>DQA1*03:01/DQB1*03:02<br>DQA1*04:01/DQB1*04:02	LRGVEEIAL<br>EEIALWLEV	2	B*39:01<br>B*40:02	

[1] Region of the sequence that is covered by overlapping 9-mers is colored in blue.

Table S3. Peptides from previously published studies applying reverse vaccinology strategy to pathogenic *Leishmania* species, used in the validation of our prediction methodology.

Predicted peptides	Viannia genomes	Viannia Topes	IEDB	IEDB matches	IEDB reported assays[1]
**Singh et al., 2015 (*L. major*)**					
SLWSLLAGV	N	N	N		
ALYTSIPVR	N	N	N		
GYPNINTYL	N	N	N		
YLLDGDQLI	N	N	N		
LYLLLPFLL	N	N	N		
GYIVVDKYF	N	N	N		
VLVGVVLGV	N	N	N		
TLFPIDVTV	N	N	N		
SFDDYTMVL	N	N	N		
VSAQTIDDY	N	N	N		
SPTPLLAAL	N	N	N		
LMAAMLVAV	N	N	N		
FLFSPTDTL	N	N	N		
HVARQLASY	N	N	N		
MLPDMTCSL	Y	Y	N		
LFLLVIYAF	N	N	N		
LFGAFLFAF	N	N	N		
AYFVVPLEM	Y	Y	N		
KHLLMAAML	N	N	N		
IHPERTVAL	Y	Y	N		
LPRLFLAFL	N	N	N		
TPRIPLDSL	N	N	N		
LVHAGIAGK	N	N	N		
AHAPQNAAL	N	N	N		
APNYYLTPL	N	N	N		
SVVDPMQNY	N	N	N		
AMKDPYTNY	N	N	N		
YLCDRTTAA	N	N	N		
LLPRLFLAF	N	N	N		
SQQETSSLY	N	N	N		
YQLTGTPVL	N	N	N		
VRAPFTIQL	Y	Y	N		
KRGAAAVLL	N	N	N		
SIMSLQIRY	Y	Y	N		
SLWSLLAGV	N	N	N		
**Agallou et al., 2014 (*L. infantum*)**					
FFAIVVTIL	Y	N	N		
HYDVSGKFA	Y	Y	N		
LYFGGVVTL	N	N	Y	LYFGGVVTLCFGLSLNHGVLVVGFNRQAKP	Agallou et al. 2014
AYVGKNGPV	N	N	N		
SALIAQTPL	N	N	N		
LYLNPNYYA	N	N	N		
NYVVTATID	N	N	N		
FFFAIVVTI	N	N	N		
QWALKNHSL	N	N	Y	GNIEGQWALKNHSLVSLSEQVLVSCDNIDD	Agallou et al. 2017<br>Agallou et al. 2014
RFNAFKQNM	Y	N	N		
GYIRLAMGS	N	N	N		
QVLVSCDNI	N	N	Y	GNIEGQWALKNHSLVSLSEQVLVSCDNIDD	Agallou et al. 2017<br>Agallou et al. 2014

QNMQTAYFL	N	N	N		
GNIEGQWAL	Y	Y	Y	GNIEGQWALKNHSLVSLSEQVLVSCDNIDD	Agallou et al. 2017<br>Agallou et al. 2014
MCGSCWAFATTGNIE	Y	N	N		
PHDEEEIAAYVGKNG	Y	N	N		
KDYKEHVHVDDSVRS	N	N	N		
GKNGPVAVAVDATTW	S	N	N		
GVDDFIASAHYGRFK	Y	N	N		
GSSWGEKGYIRLAMG	Y	N	N		
RNPFFFAIVVTILFV	S	N	N		
NPFFFAIVVTILFVV	Y	N	N		
VTILFVVCYGSALIA	S	N	N		
SLVSLSEQVLVSCDN	Y	N	Y	GNIEGQWALKNHSLVSLSEQVLVSCDNIDD	Agallou et al. 2017<br>Agallou et al. 2014
VSLSEQVLVSCDNID	Y	N	Y	GNIEGQWALKNHSLVSLSEQVLVSCDNIDD	Agallou et al. 2017<br>Agallou et al. 2014
VVTILFVVCYGSALI	Y	N	N		
GPVAVAVDATTWQLY	Y	N	N		
VTLCFGLSLNHGVLV	Y	N	Y	LYFGGVVTLCFGLSLNHGVLVVGFNRQAKP	Agallou et al. 2014
KQNMQTAYFLNAHNP	Y	N	N		
LYFGGVVTLCFGLSL	Y	N	Y	LYFGGVVTLCFGLSLNHGVLVVGFNRQAKP	Agallou et al. 2014
KPPYWIVKNSWGSSW	Y	N	N		
NQCLLKNYVVTATID	S	N	N		
SYPYTSAGGTRPPCH	Y	N	N		
KNSWGSSWGEKGYIR	Y	N	N		
KKAGAKKAV	N	N	Y	AGAKKAGAKKAVRKVATPKK	Agallou et al. 2014
SSDSAVAAL	N	N	Y	MSSDSAVAALSAAMTSPQKS	Agallou et al. 2014
MSSDSAVAALSAAMT	N	N	Y	MSSDSAVAALSAAMTSPQKS	Agallou et al. 2014
KTAAKKAAAKKAAAK	N	N	N		
KAAAKKAAAKKAGAK	N	N	N		
KAAAKKAGAKKAGAK	N	N	Y	AGAKKAGAKKAVRKVATPKK	Agallou et al. 2014
KAGAKKAGAKKAVRK	N	N	Y	AGAKKAGAKKAVRKVATPKK	Agallou et al. 2014
SSDSAVAALSAAMTS	N	N	Y	MSSDSAVAALSAAMTSPQKS	Agallou et al. 2014
KVATPKKPAKKAAKK	N	N	Y	AGAKKAGAKKAVRKVATPKK	Agallou et al. 2014
KKAAAKKAGAKKAGA	N	N	Y	AGAKKAGAKKAVRKVATPKK	Agallou et al. 2014
KKAGAKKAGAKKAVR	N	N	Y	AGAKKAGAKKAVRKVATPKK	Agallou et al. 2014
AAKKPAKKVAKKPAK	N	N	N		
SPQKSPRSSPKKTAA	S	N	N		
PRSSPKKTAAKKAAA	N	N	N		
KKTAAKKAAAKKAAA	N	N	N		
KKAAAKKAAAKKAGA	N	N	N		
AKKAGAKKAVRKVAT	N	N	Y	AGAKKAGAKKAVRKVATPKK	Agallou et al. 2014
KKAVRKVATPKKPAK	N	N	Y	AGAKKAGAKKAVRKVATPKK	Agallou et al. 2014
RKVATPKKPAKKAAK	N	N	Y	AGAKKAGAKKAVRKVATPKK	Agallou et al. 2014
TPKKPAKKAAKKAAK	N	N	N		
PAKKAAKKAAKKPAK	N	N	N		
AAKKAAKKPAKKVAK	N	N	N		
PAKKVAKKPAKKAAK	N	N	N		
VAKKPAKKAAKKPAK	N	N	N		
PAKKAAKKPAKKPAK	N	N	N		
AAKKPAKKAAKKAAK	N	N	N		
PAKKPAKKAAKKAAK	N	N	N		
PAKKAAKKAAKKAAA	N	N	N		
SAVAALSAAMTSPQK	N	N	Y	MSSDSAVAALSAAMTSPQKS	Agallou et al. 2014
AKKVAKKPAKKAAKK	N	N	N		
AKKAAKKAAKKAAAK	N	N	N		
AAKKAAAKKAAAKKA	Y	Y	N		
AKKAAAKKAAAKKAG	N	N	N		

PKKTAAKKAAAKKAA	N	N	N		
SPKKTAAKKAAAKKA	N	N	N		
SDSAVAALSAAMTSP	N	N	Y	MSSDSAVAALSAAMTSPQKS	Agallou et al. 2014
TAAKKAAAKKAAAKK	N	N	N		
AKKAAAKKAGAKKAG	N	N	Y	AGAKKAGAKKAVRKVATPKK	Agallou et al. 2014
AAKKAAAKKAGAKKA	N	N	Y	AGAKKAGAKKAVRKVATPKK	Agallou et al. 2014
DSAVAALSAAMTSPQ	N	N	Y	MSSDSAVAALSAAMTSPQKS	Agallou et al. 2014
AGAKKAVRKVATPKK	N	N	Y	AGAKKAGAKKAVRKVATPKK	Agallou et al. 2014
AAAKKAAAKKAGAKK	N	N	N		
GAKKAVRKVATPKKP	N	N	Y	AGAKKAGAKKAVRKVATPKK	Agallou et al. 2014
TYEEFSAKL	N	N	Y	ATTYEEFSA, EEFSAKLDR, EFSAKLDRL, MATTYEEFS, MATTYEEFSAKLDRLDEEFNRKMQEQNAKFFADKPDES, TTYEEFSAK, TYEEFSAKL, YEEFSAKLD, MATTYEEFAAKLDRLDEEFN, TYEEFSAKLDRLDEEFNRKM	Agallou et al. 2014<br>Basu et al.2007<br>Delgado et al. 2003<br>Jensen et al. 1998
HYEKFERMI	Y	Y	Y	AKFFADKPDESTLSPEMREHYEKFERMIKEHTEKFNKK, EHYEKFERM, EKFERMIKE, HYEKFERMI, KFERMIKEH, MREHYEKFE, REHYEKFER, YEKFERMIK, EHYDKFERMIKEHTEKFNKK	Basu et al. 2007<br>Delgado et al. 2003<br>Jensen et al. 1998
HFKQFAEL	N	N	N		
HSEHFKQKF	N	N	Y	EHFKQKFAE, EHSEHFKQK, HEHSEHFKQ, HFKQKFAEL, HSEHFKQKF, IKEHTEKFNKKMHEHSEHFKQKFAELLEQQKAAQYPSK, MHEHSEHFK, SEHFKQKFA, KFNKKMHEHSEHFKHKFAEL	Basu et al. 2007<br>Delgado et al. 2003<br>Jensen et al. 1998
EFSAKLDRL	N	N	Y	AKLDRLDEE, EEFSAKLDR, EFSAKLDRL, FSAKLDRLD, MATTYEEFSAKLDRLDEEFNRKMQEQNAKFFADKPDES, SAKLDRLDE, TYEEFSAKL, YEEFSAKLD, TYEEFSAKLDRLDEEFNRKM, MATTYEEFAAKLDRLDEEFN	Agallou et al. 2014<br>Basu et al. 2007<br>Delgado et al. 2003<br>Jensen et al. 1998
TYEEFSAKLDRLDEE	Y	N	Y	AKLDRLDEE, ATTYEEFSA, DRLDEEFNR, EEFSAKLDR, EFSAKLDRL, FSAKLDRLD, KLDRLDEEF, LDRLDEEFN, MATTYEEFS, MATTYEEFSAKLDRLDEEFNRKMQEQNAKFFADKPDES, SAKLDRLDE, TTYEEFSAK, TYEEFSAKL, YEEFSAKLD (donovani); TYEEFSAKLDRLDEEFNRKM, MATTYEEFAAKLDRLDEEFN	Agallou et al. 2014<br>Basu et al. 2007<br>Delgado et al. 2003<br>Jensen et al. 1998
QKFAELLEQQKAAQN	S	N	Y	AELLEQQKA, ELLEQQKAA, EQQKAAQYP, FAELLEQQK, FKQKFAELL, HFKQKFAEL, IKEHTEKFNKKMHEHSEHFKQKFAELLEQQKAAQYPSK, KFAELLEQQ, KQKFAELLE, LEQQKAAQY, LLEQQKAAQ, QKFAELLEQ, QQKAAQYPS, FKHKFAELLEQQKAAQYPSK, KFNKKMHEHSEHFKHKFAEL	Basu et al. 2007<br>Delgado et al. 2003<br>Jensen et al. 1998
KEHYEKFERMIKEHT	Y	Y	Y	AKFFADKPDESTLSPEMREHYEKFERMIKEHTEKFNKK, EHYEKFERM, EKFERMIKE, EMREHYEKF, ERMIKEHTE, FERMIKEHT, HYEKFERMI, IKEHTEKFN, KEHTEKFNK, KFERMIKEH, MIKEHTEKF, MREHYEKFE, PEMREHYEK, REHYEKFER, RMIKEHTEK, YEKFERMIK, EHYDKFERMIKEHTEKFNKK, FADKPDESTLSPEMKEHYDK	Basu et al.2007<br>Delgado et al. 2003<br>Jensen et al. 1998
KQKFAELLEQQKAAQ	Y	N	Y	AELLEQQKA, EHFKQKFAE, ELLEQQKAA, EQQKAAQYP, FAELLEQQK, FKQKFAELL, HFKQKFAEL, IKEHTEKFNKKMHEHSEHFKQKFAELLEQQKAAQYPSK, KFAELLEQQ, KQKFAELLE, LEQQKAAQY, LLEQQKAAQ, QKFAELLEQ, QQKAAQYPS, FKHKFAELLEQQKAAQYPSK, KFNKKMHEHSEHFKHKFAEL	Basu et al.2007<br>Delgado et al. 2003<br>Jensen et al. 1998
HYHTQIDEL	Y	Y	Y	VTEKDVELLHEIEAHYHTQIDELPVDFAAY	Agallou et al. 2014
RYGRKGVAI	Y	Y	N		
APQDQDSFL	Y	N	Y	KIAPQDQDSFLDDQPGVRPIPSFDDMPLHQ	Agallou et al. 2014

IPSFDDMPL	Y	Y	Y	FLDDQPGVRPIPSFDDM,<br>KIAPQDQDSFLDDQPGVRPIPSFDDMPLHQ	Agallou et al. 2014<br>Koutsoni et al. 2017
LPKDIQVAL	Y	Y	Y	PKDIQVALFSATMPEEVL,<br>DEMLSQGFADQIYEIFRFLPKDIQVALFSA	Agallou et al. 2014<br>Koutsoni et al. 2017
LPTNKENYL	Y	N	N		
LPVDFAAYL	Y	Y	Y	VTEKDVELLHEIEAHYHTQIDELPVDFAAY	Agallou et al. 2014
SFLDDQPGV	Y	N	Y	FLDDQPGVRPIPSFDDM;<br>KIAPQDQDSFLDDQPGVRPIPSFDDMPLHQ	Agallou et al. 2014<br>Koutsoni et al. 2017
RFLPKDIQV	Y	Y	Y	PKDIQVALFSATMPEEVL<br>DEMLSQGFADQIYEIFRFLPKDIQVALFSA	Agallou et al. 2014<br>Koutsoni et al. 2017
LSPTRELAL	Y	Y	Y	TRELALQTAEVISRIGE	Koutsoni et al. 2017
GFADQIYEI	Y	Y	Y	DEMLSQGFADQIYEIFRFLPKDIQVALFSA	Agallou et al. 2014
SRVLVTTDL	Y	Y	N		
RPIPSFDDM	Y	Y	Y	FLDDQPGVRPIPSFDDM,<br>KIAPQDQDSFLDDQPGVRPIPSFDDMPLHQ	Agallou et al. 2014<br>Koutsoni et al. 2017
NSSKFCETF	Y	N	N		
VSIAQSVIF	Y	Y	N		
KFMRDPVRI	Y	Y	N		
IFRFLPKDI	Y	N	Y	DEMLSQGFADQIYEIFRFLPKDIQVALFSA	Agallou et al. 2014
IFANTRRKV	Y	Y	N		
TFRSGSSRV	Y	Y	N		
RGALRTESL	Y	Y	N		
LSPTRELALQTAEVI	Y	Y	Y	TRELALQTAEVISRIGE	Koutsoni et al. 2017
VLVTTDLVARGICVH	Y	N	N		
LPKDIQVALFSATMP	Y	N	Y	PKDIQVALFSATMPEEVL,<br>DEMLSQGFADQIYEIFRFLPKDIQVALFSA	Agallou et al. 2014<br>Koutsoni et al. 2017
HTQIDELPVDFAAYL	Y	Y	Y	VTEKDVELLHEIEAHYHTQIDELPVDFAAY	Agallou et al. 2014
QDDLRKLQAGVIVAV	Y	N	N		
ALRTESLRVLVLDEA	Y	Y	N		
TESLRVLVLDEADEM	Y	Y	N		
RGGDIIAQAQSGTGK	Y	Y	Y	IQQRAIAPFTRGGDII	Koutsoni et al. 2017
DLRKLQAGVIVAVGT	Y	N	N		
RKLQAGVIVAVGTPG	Y	N	N		
MDLYETVSIAQSVIF	Y	Y	N		
FMRDPVRILVKRESL	Y	Y	N		
DLVARGIDVHHVNIV	Y	N	N		
RTESLRVLVLDEADE	Y	Y	N		
ETVSIAQSVIFANTR	Y	N	N		
TVSSMHAEMPKSDRE	Y	N	N		
RSGSSRVLVTTDLVA	Y	N	N		
AQSVIFANTRRKVDW	Y	Y	N		
LDDQPGVRPIPSFDD	Y	N	Y	FLDDQPGVRPIPSFDDM,<br>KIAPQDQDSFLDDQPGVRPIPSFDDMPLHQ	Agallou et al. 2014<br>Koutsoni et al. 2017
QSGTGKTGAFSIGLL	Y	Y	N		
ALQTAEVISRIGEFL	Y	Y	Y	TRELALQTAEVISRIGE	Koutsoni et al. 2017
VLVLDEADEMLSQGF	Y	Y	Y	DEMLSQGFADQIYEIFRFLPKDIQVALFSA	Agallou et al. 2014
LDTLMDLYETVSIAQ	Y	Y	N		
VELLHEIEAHYHTQI	Y	N	Y	VTEKDVELLHEIEAHYHTQIDELPVDFAAY	Agallou et al. 2014
TGAFSIGLLQRLDFR	Y	Y	N		
SIGLLQRLDFRHNLI	Y	N	N		
VSDVIKRGALRTESL	Y	Y	N		
PTRELALQTAEVISR	Y	Y	Y	TRELALQTAEVISRIGE	Koutsoni et al. 2017
TRELALQTAEVISRI	Y	Y	Y	TRELALQTAEVISRIGE	Koutsoni et al. 2017
SPTRELALQTAEVIS	Y	Y	Y	TRELALQTAEVISRIGE	Koutsoni et al. 2017
DDLRKLQAGVIVAVG	Y	N	N		
VLSPTRELALQTAEV	Y	Y	Y	TRELALQTAEVISRIGE	Koutsoni et al. 2017
LVLSPTRELALQTAE	Y	y	Y	TRELALQTAEVISRIGE	Koutsoni et al. 2017
LRKLQAGVIVAVGTP	Y	N	N		
PSSIQQRAIAPFTRG	Y	Y	Y	IQQRAIAPFTRGGDII, KPSSIQQRAIAPFTRGG	Koutsoni et al. 2017

KPSSIQQRAIAPFTR	Y	Y	Y	IQQRAIAPFTRGGDII, KPSSIQQRAIAPFTRGG	Koutsoni et al. 2017
SSIQQRAIAPFTRGG	Y	Y	Y	IQQRAIAPFTRGGDII, KPSSIQQRAIAPFTRGG	Koutsoni et al. 2017
GLVLSPTRELALQTA	Y	N	Y	TRELALQTAEVISRIGE	Koutsoni et al. 2017
SNHTVSSMHAEMPKS	Y	N	N		
**Naouar et al., 2014 (*L. major*)**					
ALQEETHVL	N	N	Y	ALQEETHVL	Naouar et al. 2014
YMAQKAEEV	Y	Y	Y	YMAQKAEEV	Naouar et al. 2014
KLTVSSAAV	N	N	Y	KLTVSSAAV	Naouar et al. 2014
VLGSHVQTL	N	N	Y	VLGSHVQTL	Naouar et al. 2014
LLRQETARL	N	N	Y	LLRQETARL	Naouar et al. 2014
HLMGQLNEL	Y	Y	Y	HLMGQLNEL	Naouar et al. 2014
RLRDDLERL	N	N	Y	RLRDDLERL	Naouar et al. 2014
YLLDVSTLL	Y	Y	Y	YLLDVSTLL	Naouar et al. 2014
NLIDFNFKL	Y	Y	Y	NLIDFNFKL	Naouar et al. 2014
LLKDSFAFL	Y	Y	Y	LLKDSFAFL	Naouar et al. 2014
CLLDSFKEL	Y	Y	Y	CLLDSFKEL	Naouar et al. 2014
VLEENRTTL	Y	Y	Y	VLEENRTTL	Naouar et al. 2014
VLCALLFCV	N	N	Y	VLCALLFCV	Naouar et al. 2014
KLHPVYDKV	N	N	Y	KLHPVYDKV	Naouar et al. 2014
ALKGSLVAV	N	N	N		Naouar et al. 2014
EMASMITKV	N	N	Y	EMASMITKV	Naouar et al. 2014
DMLAGIATL	N	N	Y	DMLAGIATL	Naouar et al. 2014
LLSAQIARL	Y	Y	Y	LLSAQIARL	Naouar et al. 2014
LLFDELTAL	Y	Y	Y	LLFDELTAL	Naouar et al. 2014
RLMQCVQQL	N	N	Y	RLMQCVQQL	Naouar et al. 2014
SLVVVSASL	N	N	Y	SLVVVSASL	Naouar et al. 2014
SLCRSLVVV	N	N	Y	SLCRSLVVV	Naouar et al. 2014
HLVAPLASL	N	N	Y	HLVAPLASL	Naouar et al. 2014
ALNDALWAV	Y	Y	Y	ALNDALWAV	Naouar et al. 2014
RLLVDLAQL	Y	Y	Y	RLLVDLAQL	Naouar et al. 2014
IVVDAIMSV	N	N	Y	IVVDAIMSV	Naouar et al. 2014
VIAGTSNAV	N	N	Y	VIAGTSNAV	Naouar et al. 2014
AMREALRYL	Y	Y	Y	AMREALRYL	Naouar et al. 2014
GVFDAAISI	Y	Y	Y	GVFDAAISI	Naouar et al. 2014
QVGAFLEGL	N	N	Y	QVGAFLEGL	Naouar et al. 2014
GLDYSEELL	Y	N	Y	GLDYSEELL	Naouar et al. 2014
RVAASVAAV	N	N	Y	RVAASVAAV	Naouar et al. 2014
GTDDTVAAV	N	N	Y	GTDDTVAAV	Naouar et al. 2014
TIPSFIVRV	Y	Y	Y	TIPSFIVRV	Naouar et al. 2014
RLLEGSAIM	Y	Y	Y	RLLEGSAIM	Naouar et al. 2014
LIQQRHIAV	N	N	Y	LIQQRHIAV	Naouar et al. 2014
AVAKQIVTI	Y	Y	Y	AVAKQIVTI	Naouar et al. 2014
ILERRLQTI	N	N	Y	ILERRLQTI	Naouar et al. 2014
NMMAVVGLL	N	N	Y	NMMAVVGLL	Naouar et al. 2014
KLEDEVFAL	N	N	Y	KLEDEVFAL	Naouar et al. 2014
ELLGNLEEV	N	N	Y	ELLGNLEEV	Naouar et al. 2014
RMADEVQRL	N	N	Y	RMADEVQRL	Naouar et al. 2014
RLAVSLHEL	N	N	Y	RLAVSLHEL	Naouar et al. 2014
LLGPAYQSI	N	N	Y	LLGPAYQSI	Naouar et al. 2014
VIAEEPLYV	N	N	Y	VIAEEPLYV	Naouar et al. 2014
PLSAVISPV	Y	Y	Y	PLSAVISPV	Naouar et al. 2014
LLPAPLVSV	N	N	Y	LLPAPLVSV	Naouar et al. 2014
MLLWTAVAV	N	N	Y	MLLWTAVAV	Naouar et al. 2014
YLRTFPAAL	Y	Y	Y	YLRTFPAAL	Naouar et al. 2014
RLAGFLAGL	N	N	Y	RLAGFLAGL	Naouar et al. 2014
CLALIAWRV	N	N	Y	CLALIAWRV	Naouar et al. 2014
VVAGMLRWV	N	N	Y	VVAGMLRWV	Naouar et al. 2014

PLSPATRRL	N	N	Y	PLSPATRRL	Naouar et al. 2014
MVLNAMAWL	N	N	Y	MVLNAMAWL	Naouar et al. 2014
KIMEAITVV	N	N	Y	KIMEAITVV	Naouar et al. 2014
FLAYDKFLL	Y	Y	Y	FLAYDKFLL	Naouar et al. 2014
HIFDRVAGV	N	N	Y	HIFDRVAGV	Naouar et al. 2014
ALNQFTKVL	Y	N	Y	ALNQFTKVL	Naouar et al. 2014
AIVKDMARL	N	N	Y	AIVKDMARL	Naouar et al. 2014
GLQEVTRAI	N	N	Y	GLQEVTRAI	Naouar et al. 2014
VIANNVDPV	Y	Y	Y	VIANNVDPV	Naouar et al. 2014
TLKNLIRSV	N	N	Y	TLKNLIRSV	Naouar et al. 2014
TLLDALGML	N	N	Y	TLLDALGML	Naouar et al. 2014
ALLAFTLGV	Y	Y	Y	ALLAFTLGV	Naouar et al. 2014
TLGVKQMVV	N	N	Y	TLGVKQMVV	Naouar et al. 2014
KLVRELFRV	Y	Y	Y	KLVRELFRV	Naouar et al. 2014
TMLELLTQL	Y	Y	Y	TMLELLTQL	Naouar et al. 2014
ALRERRMKV	N	N	Y	ALRERRMKV	Naouar et al. 2014
LLHDRQHSI	Y	Y	Y	LLHDRQHSI	Naouar et al. 2014
GLEQQIQEI	Y	N	Y	GLEQQIQEI	Naouar et al. 2014
MLQTNSLAL	N	N	Y	MLQTNSLAL	Naouar et al. 2014
SLQFSAFLL	N	N	Y	SLQFSAFLL	Naouar et al. 2014
FMEVFGMLV	N	N	Y	FMEVFGMLV	Naouar et al. 2014
MLVQSCTSI	N	N	Y	MLVQSCTSI	Naouar et al. 2014
VVSVLTHSV	N	N	Y	VVSVLTHSV	Naouar et al. 2014
WIPPVVSVL	N	N	Y	WIPPVVSVL	Naouar et al. 2014
KIYQIGRSV	N	N	Y	KIYQIGRSV	Naouar et al. 2014
QLNKKIYQI	Y	N	Y	QLNKKIYQI	Naouar et al. 2014
**Dikhit et al., 2017 (*L. donovani*)**					
QSKFRPISASSMPDE	N	N	N		
IQSYRLLKDAVASPL	N	N	N		
LQSFVVTCSAAALTL	N	N	N		
QSFVVTCSAAALTLA	N	N	N		
ALLQSFVVTCSAAAL	S	N	N		
ILDVIFMTSRLVAKM	Y	N	N		
LRRLFSIRTNALARE	Y	N	N		
FDLFLFSNGAVVWWG	Y	Y	Y	FDLFLFSNGAVVWWG	Dikhit et al. 2017
TLGFQPLAVEPALDR	S	N	N		
NCFDLFLFSNGAVVW	Y	N	Y	FDLFLFSNGAVVWWG	Dikhit et al. 2017
RVRLRRLFSIRTNAL	Y	N	N		
LLALILLGGIGAVGY	N	N	N		
YPVYPFLASNAALLN	Y	N	Y	VYPFLASNAALLNLI, YPVYPFLASNAALLN	Dikhit et al. 2017
LPSFHAMSAFHSAAK	N	N	N		
NAALLNLIPSLLYRV	S	N	Y	VYPFLASNAALLNLI, YPVYPFLASNAALLN	Dikhit et al. 2017
VYPFLASNAALLNLI	Y	N	Y	VYPFLASNAALLNLI, YPVYPFLASNAALLN	Dikhit et al. 2017
RALLALILLGGIGAV	N	N	N		
AALLNLIPSLLYRVQ	Y	N	Y	VYPFLASNAALLNLI	Dikhit et al. 2017
LQVFTAICASFAHGA	Y	Y	N		
FPFFSGVAPIVASWF	Y	Y	N		
ERVFRYLQVFTAICA	Y	N	N		
KDDFPFFSGVAPIVA	Y	N	N		
DFPFFSGVAPIVASW	Y	N	N		
LESFFVLFKGASKRL	Y	Y	N		
FFVLFKGASKRLKWS	Y	Y	N		
FRYLQVFTAICASFA	Y	N	N		
SRGFSAELSAALVVS	Y	Y	N		
RGFSAELSAALVVSF	Y	Y	N		
LALLIMLYALIATQF	N	N	Y	IMLYALIATQFSDDA, LALLIMLYALIATQF, LIMLYALIATQFSDD	Dikhit et al. 2017

LIMLYALIATQFSDD	N	N	Y	IMLYALIATQFSDDA, LALLIMLYALIATQF, LIMLYALIATQFSDD	Dikhit et al. 2017
IMLYALIATQFSDDA	N	N	Y	IMLYALIATQFSDDA, LALLIMLYALIATQF, LIMLYALIATQFSDD	Dikhit et al. 2017
VSVLALLIMLYALIA	N	N	Y	IMLYALIATQFSDDA, LALLIMLYALIATQF, LIMLYALIATQFSDD	Dikhit et al. 2017
VLALLIMLYALIATQ	N	N	Y	IMLYALIATQFSDDA, LALLIMLYALIATQF, LIMLYALIATQFSDD	Dikhit et al. 2017
VSILRQLLSVTAHTH	Y	N	N		
KVAVSILRQLLSVTA	Y	N	N		
GISFSRAFAANIESA	Y	Y	N		
YQFYHRARSYVIFTT	Y	N	N		

[1] Reported assays:

Agallou et al. (2014): ^{3}H-thymidine proliferation, ELISA IL-10 release, ICS IFNγ release and IL-4 release / *Mus musculus* BALB/c

Agallou et al. (2017): cytometric bead array for IFNγ, TNFα, IL-2, IL-4,IL-10 release, ^{3}H-thymidine proliferation, ELISA IL-10 release, ICS IFNγ release, IL-4 release / *Mus musculus* BALB/c

Basu et al. (2007): ELISPOT IFNγ / *Homo sapiens*

Delgado et al. (2003): ^{3}H-thymidine proliferation, ELISA IFNγ release, ELISA IL-4 / *Homo sapiens*

Dikhit et al. (2017): ICS IFNγ release, ICS IL-10 release / *Homo sapiens*

Jensen et al. (1998): ^{3}H-thymidine proliferation / *Homo sapiens*

Koutsoni et al. (2017): ^{3}H-thymidine proliferation / *Mus musculus* BALB/c

Naouar et al. (2016): cellular MHC/direct/fluorescence qualitative binding / *Homo sapiens*

Table S4. Peptides with positive experimental results in IEDB, reported in studies exclusively conducted on *L. braziliensis* or *L. panamensis*.

Peptide	Species	Match in ViannaTopes	Allele coverage	Predicted alleles
AYIDGHVTI	*L. braziliensis*	AYIDGHVTI	3	HLA-A*24:02<br>HLA-C*04:01<br>HLA-C*07:02
AYLASCDFI	*L. braziliensis*	AYLASCDFI	3	HLA-A*24:02<br>HLA-C*04:01
IYVSYADLI	*L. braziliensis*	IYVSYADLI	2	HLA-A*24:02<br>HLA-C*04:01<br>HLA-C*07:02
KYQHSTEML	*L. braziliensis*	KYQHSTEML	3	HLA-A*24:02<br>HLA-C*04:01<br>HLA-C*07:02
SYMGYFQNI	*L. braziliensis*	SYMGYFQNI	3	HLA-A*24:02<br>HLA-C*04:01<br>HLA-C*07:02
TYQRVYATL	*L. braziliensis*	TYQRVYATL	3	HLA-A*24:02<br>HLA-C*04:01<br>HLA-C*07:02
VYLSFGFRL	*L. braziliensis*	VYLSFGFRL	3	HLA-A*24:02<br>HLA-C*04:01<br>HLA-C*07:02
WYLATHSLI	*L. braziliensis*	WYLATHSLI	3	HLA-A*24:02<br>HLA-C*04:01<br>HLA-C*07:02
DEEFNKKMQEQNAKFFADKP	*L. panamensis*	MQEQNAKFF	1	HLA-C*04:01
EHYDKFERMIKEHTEKFNKK	*L. panamensis*	DKFERMIKEHTEKFN	1	HLA-DRB1*08:02
FADKPDESTLSPEMKEHYDK	*L. panamensis*	DKPDESTLSPEMKEH	1	HLA-DQA1*04:01/DQB1*04:02
FKHKFAELLEQQKAAQYPSK	*L. panamensis*	HKFAELLEQQKAAQY	1	HLA-DRB1*08:02
KFNKKMHEHSEHFKHKFAEL	*L. panamensis*	MHEHSEHFKHKFAEL	1	HLA-DPA1*03:01/DPB1*04:02
MATTYEEFAAKLDRLDEEFN	*L. panamensis*	TYEEFAAKLDRLDEE	2	HLA-DQA1*03:01/DQB1*03:02<br>HLA-DQA1*04:01/DQB1*04:02
